# Structured-Defect Engineering of Hexagonal Boron Nitride for Identified Visible Single-Photon Emitters

**DOI:** 10.1021/acsnano.4c11413

**Published:** 2025-02-28

**Authors:** Tsz Wing Tang, Ritika Ritika, Mohsen Tamtaji, Hongwei Liu, Yunxia Hu, Zhenjing Liu, Patrick Ryan Galligan, Mengyang Xu, Jinghan Shen, Jun Wang, Jiawen You, Yuyin Li, GuanHua Chen, Igor Aharonovich, Zhengtang Luo

**Affiliations:** † Department of Chemical and Biological Engineering, The Hong Kong University of Science and Technology, Hong Kong SAR 999077, P. R. China; ‡ School of Mathematical and Physical Science, University of Technology Sydney, Ultimo, New South Wales 2007, Australia; § ARC Centre of Excellence for Transformative Meta-Optical Systems, Faculty of Science, University of Technology Sydney, Ultimo, New South Wales 2007, Australia; ∥ The Hong Kong Quantum AI Lab Limited, Hong Kong SAR 999077, China; ⊥ Department of Chemistry, The University of Hong Kong, Hong Kong SAR 999077, China

**Keywords:** defect structure engineering, hexagonal boron nitride, carbon doping, single-photon emitters, identified wavelength

## Abstract

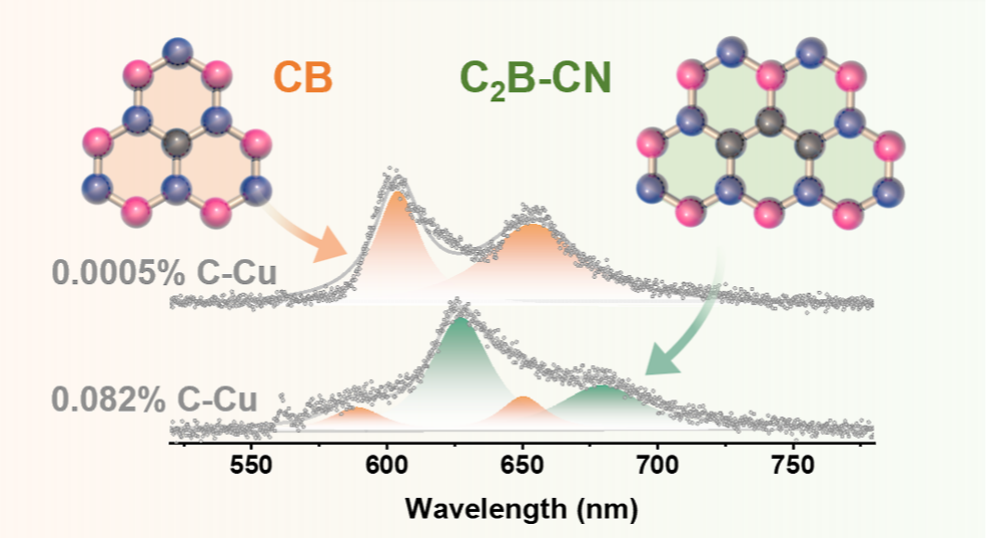

Visible-range single-photon emitters (SPEs), based on hexagonal boron nitride (hBN), with exceptional optical performance have become an outstanding candidate for quantum optical technology. However, the control of the carbon defect structures to obtain uniform and confined band structure remains elusive, restricting their integration into on-chip quantum devices. Here, we demonstrate tuning of the defect structure of hBN to precisely control the emission in SPEs. The defect structure engineering from CB (carbon substituted at the boron site) to C_2_B–CN (carbon doped into two boron sites and one nitrogen site) carbon defect conversion in hBN is realized by regulating the carbon concentration from 0.0005 at % to 0.082 at % in Cu substrates to adjust the carbon diffusion during the CVD process. Meanwhile, the zero-phonon line exhibits a precise shift from the range of 600–610 nm to 630–640 nm; these shifts of the spectral features are further supported by density functional theory results, reflected in changes in the band structure, vibrational degrees of freedom, and electronic transitions. The SPE emission spectrum serves as a valuable tool for identifying the footprint of a carbon point defect structure change. Our project offers evidence of achieving structured defect engineering for tailored emission properties and showcases potential for the integration of advanced 2D material engineering into on-chip quantum devices.

## Introduction

Recently, single-photon emitters (SPEs) have shown the capability of overcoming the limitation of traditional data transferring, communication, and computing technologies^1,2^ because of their enhanced efficiency,^3^ response time,^4^ processing rate,^5^ etc.^6^ Henceforth, SPE has become a promising candidate for quantum photonic devices, including quantum communication, quantum imaging, quantum computation, etc., attracting remarkable attention in material science. In the field of quantum communication, reliable and stable photon emitters with narrow wavelength range are necessary to enable an enormous state for quantum messaging.^7^ These SPEs with narrow wavelength ranges ensure the generation of reliable photons, facilitating efficient transmission and enabling the manipulation of quantum information in secure communication systems. However, due to the limited tunability of wavelength in existing emitter sources, researchers are actively seeking reliable single-photon sources for further development of quantum technology.

Two-dimensional (2D) materials are excellent candidates in quantum optical applications with the advantages of stretchability, heterogeneous assembly, and enhanced photonic integrated circuits.^8,9^ Among them, hexagonal boron nitride (hBN) is a promising quantum emitter due to its high single-photon purity (about g^(2)^(0) = 0.077),^10^ high thermal stability (up to 1100 K),^11^ tunable emission wavelength (from ultraviolet (UV) to near-infrared (IR) region),^12^ ultrafast repetition rate (up to megahertz),^10^ tunable emission energy (up to 6 meV),^10^ etc., providing an ideal quantum technology platform. The point defects in hBN, including vacancies and heteroatoms, are the critical emission sites of hBN-based SPEs, which create color centers and generate excellent single photonic responses.^13−19^ Recently, ion implantation^14^ and electron beam irradiation^20^ have been explored for introducing carbon point defects into hBN to generate deep-level bands within the energy band structure,^21−23^ introducing active sites responsible for photon emission within the visible range at cryogenic temperatures. Furthermore, the electronic structure of the hBN crystal with substitutional carbon atoms is found to be localized, and the shift of energy levels of the conduction band and valence band depends on the defect structure of the substitutional carbon atom in the lattice and its interaction with neighboring atoms.^24^ However, the pursuit of structured carbon defects in hBN to customize emission characteristics and comprehend the underlying emission mechanisms, along with the correlation between point defect structures and photoluminescent properties, poses significant challenges. These challenges are crucial to address future applications of on-chip quantum photonic devices. To surmount the existing challenges and limitations and fully exploit the potential of hBN as an optimized material for SPEs, it is imperative to enhance the controllability of defect structures and attain a uniform distribution, encompassing wavelength tunability and a high purity thereof. This will enable the customization of quantum photonic properties in SPEs, thereby facilitating the realization of hBN’s complete capabilities in optimized quantum devices.

In this work, we proposed a strategy to modulate wavelength in SPEs through the engineering of a carbon defect in hBN. By employing molten copper substrates during CVD synthesis, a precise control over the carbon defect structure is achieved, allowing for effective modification of carbon introduction in hBN through controlled concentration. This approach ensures a homogeneous and continuous carbon supply prior to the formation of flawless hBN flakes, resulting in an effective high level of carbon doping while simultaneously maintaining a uniform distribution of emission sites. The defect structure from CB to C_2_B–CN in hBN is realized by manipulating the carbon concentration within the range of 0.0005 at % to 0.082 at % in Cu substrates. The molten copper substrates not only provide carbon heteroatoms into hBN but also ensure a high degree of uniformity of the carbon distribution and active emission sites in synthesized hBN. Furthermore, a precise wavelength shift in the zero-phonon line (ZPL) of SPEs from the 600–610 to 630–640 nm range has been achieved. The hBN-based SPEs, boasting tailored emission wavelengths and large-scale uniformity, demonstrate significant promise in visible quantum photonics applications, paving the way for practical on-chip quantum photonic devices and offering advancements in quantum key distribution and high-resolution sensing technologies.

## Results and Discussion

### Regulation of Carbon Defect Structures in hBN

**Figure 1:**
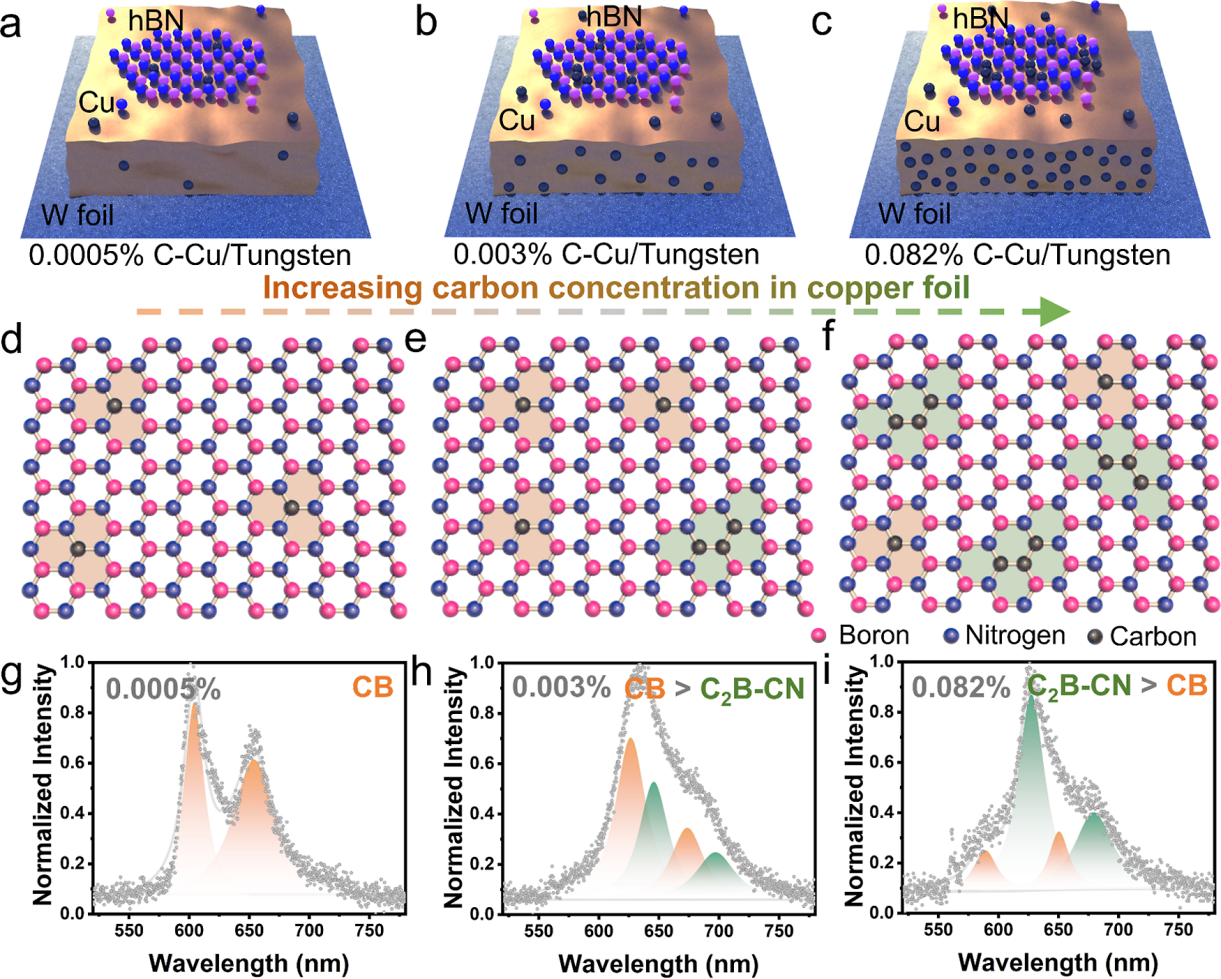
Tailoring carbon defect structures in hBN for tunable SPE wavelength. The illustration diagrams of substrate modification method for hBN defect engineering and the atomic models of various types of carbon defects in hBN formed from (a,d) 0.0005%, (b,e) 0.003%, and (c,f) 0.082% carbon-containing Cu foils. (g–i) Spectra for localized SPEs with ZPL and PSB, recorded from various hBN samples transferred on SiO_2_/Si from Cu foils with different carbon-containing concentrations, (g) 0.0005%, (h) 0.003%, and (i) 0.082%.

The engineering of carbon-doped defects in hBN by substrate modification techniques has been undertaken to explore. The investigation involved the use of Cu foils with varying carbon concentrations, 0.0005%, 0.003%, and 0.082%, to meticulously control the diffusion rates of carbon, as shown in Figure 1a–c, respectively. The respective carbon contents are accurately determined through inductively coupled plasma mass spectrometry (ICP–MS), with results depicted in Table S1. Dissolved carbon atoms in the Cu foils diffuse to the surface due to the chemical potential difference, with an enhanced diffusion rate occurring when the Cu substrates reach a molten state at 1090 °C.^25^ Meanwhile, owing to the comparable atomic size and strong chemical compatibility between carbon atoms and the constituent elements of boron and nitrogen in hBN, it is anticipated that the diffused carbon would readily integrate into the hBN lattice.^26,27^ The carbon-containing molten copper gently and continuously releases carbon during the CVD hBN growth process, ensuring the avoidance of competitive graphene growth and facilitating defect-free transitions with high-carbon copper utilization. Consequently, varying levels of carbon heteroatom doping within the hBN structure have been investigated. Notably, an increase in the carbon concentration within the Cu substrate leads to a higher quantity of carbon atoms being incorporated into the hBN lattice, resulting in the conversion of defect types in the hBN lattice, as illustrated in Figure 1d–f.

The change in defect structure within carbon-doped hBN has a significant impact on the characteristics of the SPEs, with both the ZPL and the phonon sideband (PSB), corresponding to the emission of a single photon resulting from phonon interactions in carbon-doped hBN. The SPE spectra of increasing carbon doping level are found to be associated with the increasing peak widths, while the peak positions of ZPL shift from the 600–610 nm range to 630–640 nm (Figure 1g–i). This broadening of PL peak width indicates the existence of the carbon defect structure transformation within the hBN lattice.^12,18,28,29^ In particular, the predominant defect type at lower carbon doping levels is denoted as CB (carbon substitutionally doped in the boron site). As the carbon doping level increases, the emergence of a different carbon defect structure known as C_2_B–CN (trimer defects, where carbon atoms are doped into two boron sites and one nitrogen site) takes place. This defect structure transition is expected to be closely associated with the band structure of the hBN material. In particular, the C_2_B–CN defect exhibits a lower bandgap compared to the CB defect resulting in the PL peak positions of the hBN with the C_2_B–CN defect shifting toward the higher wavelength region; the detailed explanations will be described in the subsequent sections. Our results demonstrate wavelength modification, highlighting the versatility of our method in engineering hBN defect structures for tailored SPEs.

### Structural Analysis of Carbon Incorporation into hBN Lattice

**Figure 2:**
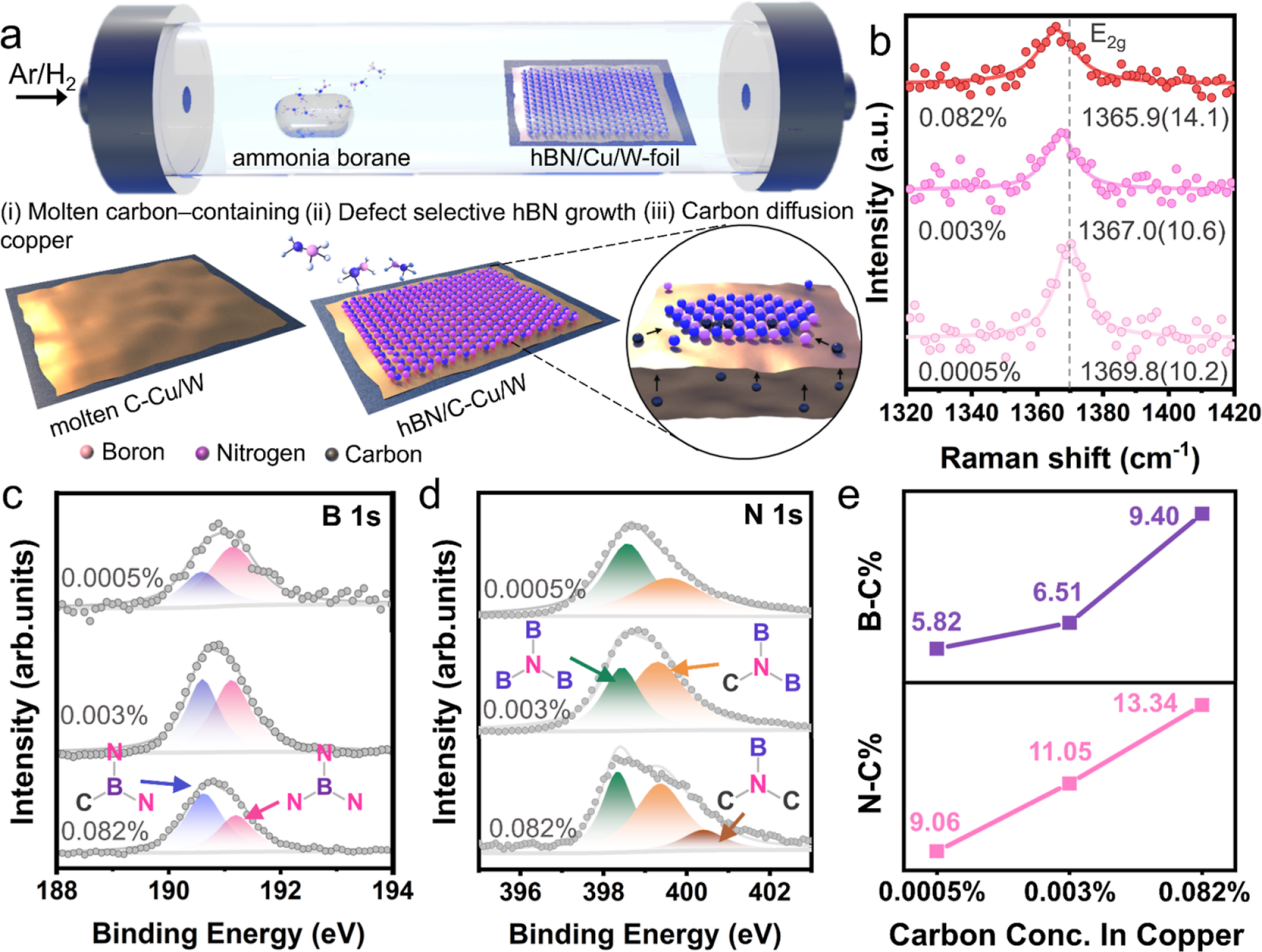
APCVD growth of carbon-doped hBN and structural characterization. (a) Illustration of the CVD setup for the carbon-doped hBN growth, employing ammonia borane as the precursor and tungsten foil-supported Cu as the substrate, and the schematic of the CVD growth processes of carbon-doped hBN. (b) Raman spectra fitted with Lorentzian line shape of the monolayer carbon-doped hBN grown from Cu foils with different carbon-containing concentrations, 0.0005% (pale pink), 0.003% (purple), and 0.082% (red), with peak positions and fwhm (in brackets) and transferred onto the SiO_2_/Si substrate. XPS spectra of (c) B 1s and (d) N 1s core levels of various hBN samples with multiple peaks fitted using Lorenz functions. The gray dots represent the raw data. (e) Percentages of B–C bonding and N–C bonding derived from the B 1s and N 1s XPS spectra after preforming multipeak optimization.

The growth of carbon-doped hBN is achieved by using an atmospheric pressure chemical vapor deposition (APCVD) system, as illustrated in Figures 2a and S1. The setup comprises two distinct heating zones. Zone 1 accommodates the ammonia borane (AB) precursor responsible for hBN growth, while zone 2 contains an electrochemically polished Cu foil stacked on a tungsten foil support as the catalytic substrate. Within 40 min, zone 2 is gradually ramped up to 1090 °C, promoting the formation of a completely melted copper layer supported by the tungsten foil. In parallel, the temperature of zone 1 is heated to 90 °C for initiating the thermal dehydration of the AB precursor, after reaching the desired growth temperature. Meanwhile, carbon impurities are introduced into the hBN lattice through the molten Cu during growth. At the same time, APCVD system offers a relatively thermodynamically stable environment for carbon incorporation, leading to the formation of uniformly distributed carbon defects to lay a cornerstone for the carbon-defect engineering of hBN. Subsequent to the growth process, the samples are placed on the hot plate and heated to 200 °C and held for 10 min. This step oxidizes the exposed copper and visualizes the as-grown hBN due to its exceptional resistance to oxygen.^30−32^ To investigate the structure and composition of the carbon-doped hBN, we transferred the monolayer hBN onto a SiO_2_/Si substrate by the bubbling transfer method.^13^

The growth of multilayer hBN hinges on the dissolution of boron and nitrogen atoms within the metal catalyst, followed by their segregation to form hBN layers.^31,33,34^ This dissolution-segregation mechanism elucidates the layer-by-layer growth of hBN from bottom to up.^34−36^ Consequently, the presence of carbon defects is promoted in each hBN layer by utilizing a carbon-dissolved copper substrate. In addition, this growth process is facilitated by the reduction of the boron oxide layer and therefore promotes the diffusion of B and N into the metal. The addition of carbon to the system introduces a variable factor that alters the kinetics of hBN growth.^37^ As a result, the amount of carbon introduced into the system prompts the nucleation and growth dynamics of hBN layers, leading to the variations in their flake shapes and morphology (Figure S2). The atomic force microscopy (AFM) results of the as-grown monolayer hBN samples are shown in Figure S3. Figure 2b shows the Raman spectra of the as-grown monolayer hBN samples fitted with a Lorentzian line shape. The peaks of those three samples are located near 1370 cm^–1^, corresponding to the *E*_2g_ in-plane vibrational mode.^38,39^ The peak position of hBN grown from 0.0005% C–Cu at the low carbon doping level is located at 1369.8 cm^–1^, showing a slight red shift for higher carbon doping to 1367.0 and 1365.9 cm^–1^, for the hBN grown from 0.003% C–Cu and 0.082% C–Cu, respectively. This shifting is attributed to the change in the lattice vibration of hBN by the incorporation of carbon heteroatoms. Moreover, the Raman peak position shifts to the left with increasing carbon content, suggesting a decrease in the vibrational frequency, in agreement with the SPE results. Furthermore, SPE spectra show that the ZPL-PSB vibration decreases from 0.1988 eV (CB) to 0.1512 eV (C_2_B–CN) by increasing the carbon content from 0.0005% C–Cu to 0.082% C–Cu. This can be justified based on higher atomic mass of carbon atoms than boron atoms, which leads to less vibrational frequency and narrowing the ZPL-PSB. Moreover, since carbon’s outer shell has more electrons than that of boron, it can make stronger bonds with nitrogen atoms inside the structure.

The crystallinity of hBN is determined by analyzing the full width at half-maximum (fwhm) obtained from the Raman results. The fwhm is 10.2 cm^–1^ for the sample from 0.0005% C–Cu and increases to 10.6 and 14.1 cm^–1^ for 0.003% and 0.082% C–Cu, respectively. Remarkably, hBN with the lowest doping level derived from 0.0005% C–Cu hBN growth demonstrated the highest level of crystallinity. This observed trend in decreasing the crystallinity of hBN as the carbon concentration rises aligns with the anticipated impact of higher doping levels of heteroatoms in the lattice. The monolayered hBN, exfoliated from bulk and free of impurities, showcases a pristine lattice structure, displaying a sharp *E*_2g_ Raman peak at 1370.6 cm^–1^ with a narrow linewidth of 9.73 cm^–1^ (fwhm), as shown in Figure S4. Furthermore, it underscores the Raman shifts in carbon-incorporated samples compared with hBN with high structural purity.

To assess the doping concentration and chemical bonding in carbon-doped hBN, X-ray photoelectron spectroscopy (XPS) is used to evaluate the doping level. The chemical bonding of hBN films is investigated through core electron measurements at the B 1s and N 1s energy levels. Figure 2c,d shows the high-resolution XPS spectra of B 1s and N 1s core levels with multipeak fitting. Two possible chemical environments reflecting BN_3_ and CBN_2_ are attributed to peaks at 190.5 and 191.2 eV in XPS spectra of the B 1s core level. The peak at a lower binding energy (190.5 eV) is assigned to the CBN_2_ configuration, which is due to the lower electronegativity of carbon compared to nitrogen, resulting in a decreased binding energy of the B 1s peak. Likewise, the presence of multiple components in the N 1s peaks indicates the existence of two distinct chemical configurations, NB_3_ (398.4 eV) and CNB_2_ (399.2 eV).^40,41^ In the case of hBN grown from 0.082% C–Cu, a broader peak width is observed, and an additional peak is assigned (C_2_NB, 400. 4 eV). Additionally, the XPS peaks at the B 1s and N 1s core levels from the exfoliated pure hBN demonstrate narrow peaks at 191.0 and 398.5 eV, respectively, aligning closely with the binding energy levels assigned to the BN_3_ (190.5 eV) and NB_3_ (398.4 eV) chemical configurations (Figure 2c,d), as shown in Figure S4. The XPS results provide us the evidence of successful doping of carbon into the hBN lattice through the substrate modification strategy. The substitutional doping level of carbon is calculated from the fitting result, where the bonding percentage is evaluated based on the relative peak areas of the corresponding chemical configurations, as shown in Figure 2e. The determined B–C bonding percentage (B–C %) exhibits a notable increase with values of 5.82% for hBN grown from 0.0005% C–Cu, 6.51% for hBN grown from 0.003% C–Cu, and 9.40% for hBN grown from 0.082% C–Cu. Similarly, the N–C bonding percentage (N–C %) exhibits an increase from 9.06% to 11.05% and further to 13.34% for the respective growth cases, suggesting a preferential tendency for carbon doping in the boron sites rather than in the nitrogen sites.

**Figure 3:**
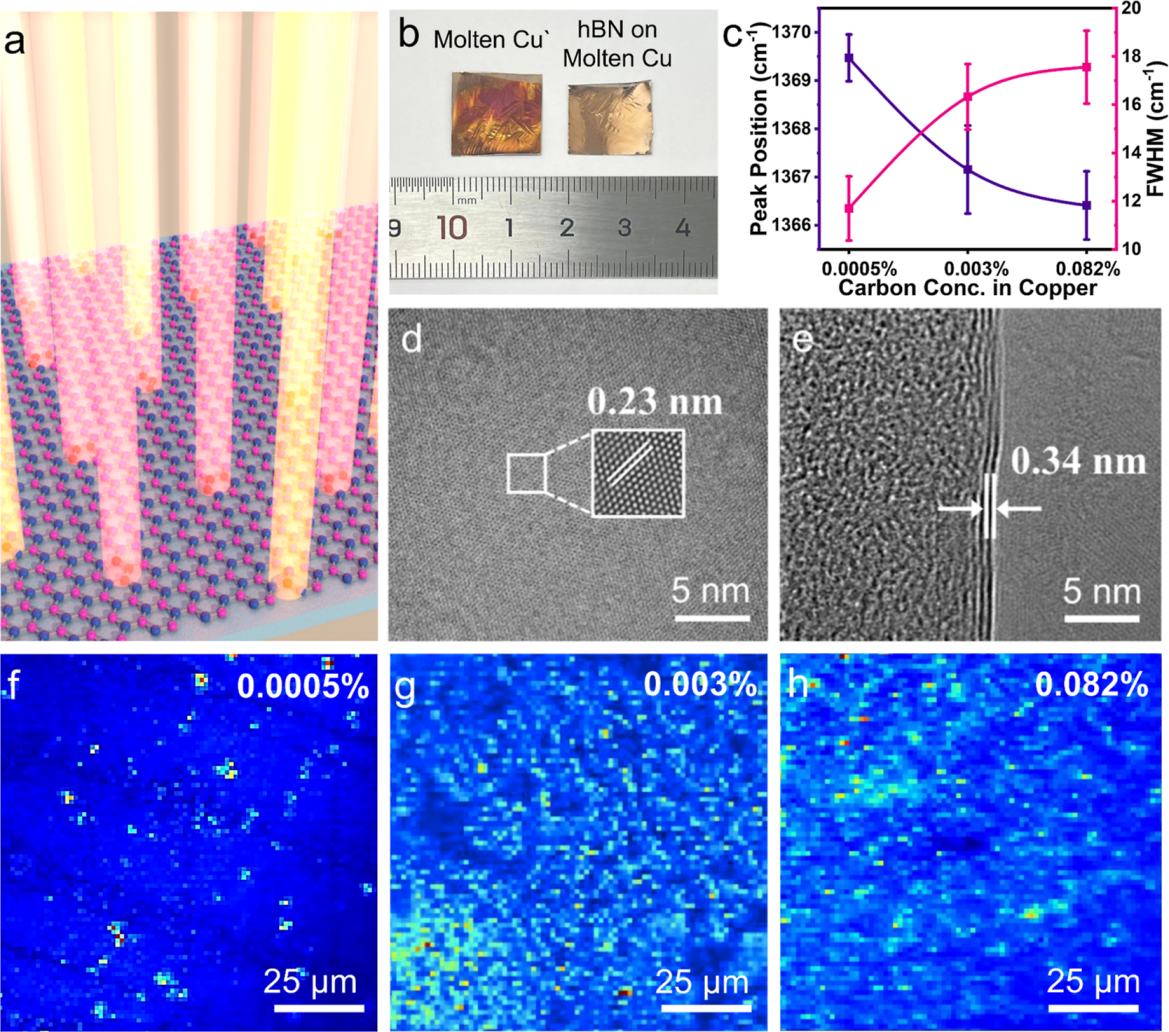
Large-scale hBN-based SPEs with highly uniform and distributed doping. (a) Schematic illustration of SPEs on active sites of carbon defects on hBN. (b) Photograph of tungsten foil-supported Cu without (left) and with (right) covered by hBN film, after heating for visualization. (c) Raman fitting results with peak positions and fwhm values obtained from 10 spots for each growth case. TEM images of hBN film grown from 0.003% carbon-containing copper, (d) *d*-spacing equals to 0.23 nm, and (e) interlayer distance equals to 0.34 nm. (f–h) Confocal scans of hBN samples grown from different carbon-containing concentrations: (f) 0.0005%, (g) 0.003%, and (h) 0.082% Cu.

The attainment of a widespread and homogeneous distribution of SPEs in hBN holds paramount importance for the progress of quantum optical technology. Hence, the implementation of precise engineering techniques to establish uniform carbon point defects within hBN samples becomes imperative. By utilizing this substrate modification, the uniformly distributed SPEs from the carbon-related active site in hBN are achieved, as shown in Figure 3a. Prolonged growth durations lead to the coalescence of hBN crystals, resulting in the subsequent formation of a complete film.^42^ Upon extending the growth time to 2.5 h, it is observed that the top surface of the Cu substrate becomes entirely coated with a layer of hBN film. Consequently, the molten Cu foil without hBN coverage undergoes oxidation, resulting in a red color after being heated at 180 °C for 2 min. In contrast, as shown in Figure 3b, the Cu foil coated with hBN retains its shining appearance due to the exceptional oxygen resistance of hBN.^31,43−45^ To substantiate the high uniformity of the SPE wavelength across the hBN films, additional direct evidence has been gathered through the collection of multiple spectra (Figure S5) from distinct regions of the same previously measured samples, demonstrating robust emission consistency with both the ZPL and PSB wavelengths, as depicted in Figure 1g–i. Investigating the uniformity of carbon incorporation in hBN, the distributions of Raman peak positions and fwhm with ten datasets are recorded and optimized for each case of hBN grown on C–Cu foils with concentrations of 0.0005%, 0.003%, and 0.082%, as shown in Figure 3c. This observed shift trend aligns with the findings discussed in Figure 2b. Moreover, the distribution of E_2g_ Raman peak positions, as measured from hBN samples grown on 0.003% C–Cu, reveals an average *E*_2g_ mode position at 1367.1 cm^–1^. The consistent peak positions observed across the surface signify uniform carbon incorporation (Figure S6). Moreover, the intensity mapping of the *E*_2g_ Raman peak for each growth case exhibited consistent and homogeneous intensity across the surface, indicating a high level of uniformity and quality of carbon defects in the samples, as shown in Figure S7. The crystal structure of the above sample was characterized by transmission electron microscopy (TEM), showing an excellent crystallinity of hBN with a clear lattice pattern (Figure 3d–e). The *d*-spacing and interlayer distance of the hBN film are 2.3 and 3.4 Å, respectively, which is similar to that of the reported values of hBN.^46^ In addition, the corresponding EDS mapping of the same hBN film grown from 0.003% carbon-containing Cu foil shows constantly distributed B, N, and C elements, as shown in Figure S8. The transferred samples from each case (Figure S9) were conducted in second-order intensity autocorrelation function g^(2)^(τ) measurements and SPE confocal scans. The measurement of g^(2)^(τ) is an important assessment for discerning the quantum characteristics of SPEs and serves as a purity metric. In quantum optics, a lower value of g^(2)^(0) indicates stronger antibunching properties, which means that the photons are more likely to be emitted one at a time rather than in pairs or groups.^1,47^ The g^(2)^(τ) results from each case confirm the quantum nature of the emitted photons, as illustrated in Figure S10. Figure 3f–h shows the PL confocal scanning of hBN films grown from each case, demonstrating a considerable increase in brightness and the emergence of isolated brilliant spots, indicating the creation of additional active sites for SPEs with higher levels of incorporated carbon in hBN. Of particular significance, the observed bright spots demonstrate a remarkably uniform distribution throughout the sample. The dark regions in the images correspond to cracks in the hBN film formed during the transfer. The presence of such uniformly distributed active sites holds crucial importance as it allows for consistent and predictable emission characteristics. This uniformity facilitates precise control and utilization of the emitted photons in diverse applications, including quantum information processing and nanophotonic.

### Mechanisms of Carbon-Doped hBN SPE Wavelength Engineering

**Figure 4:**
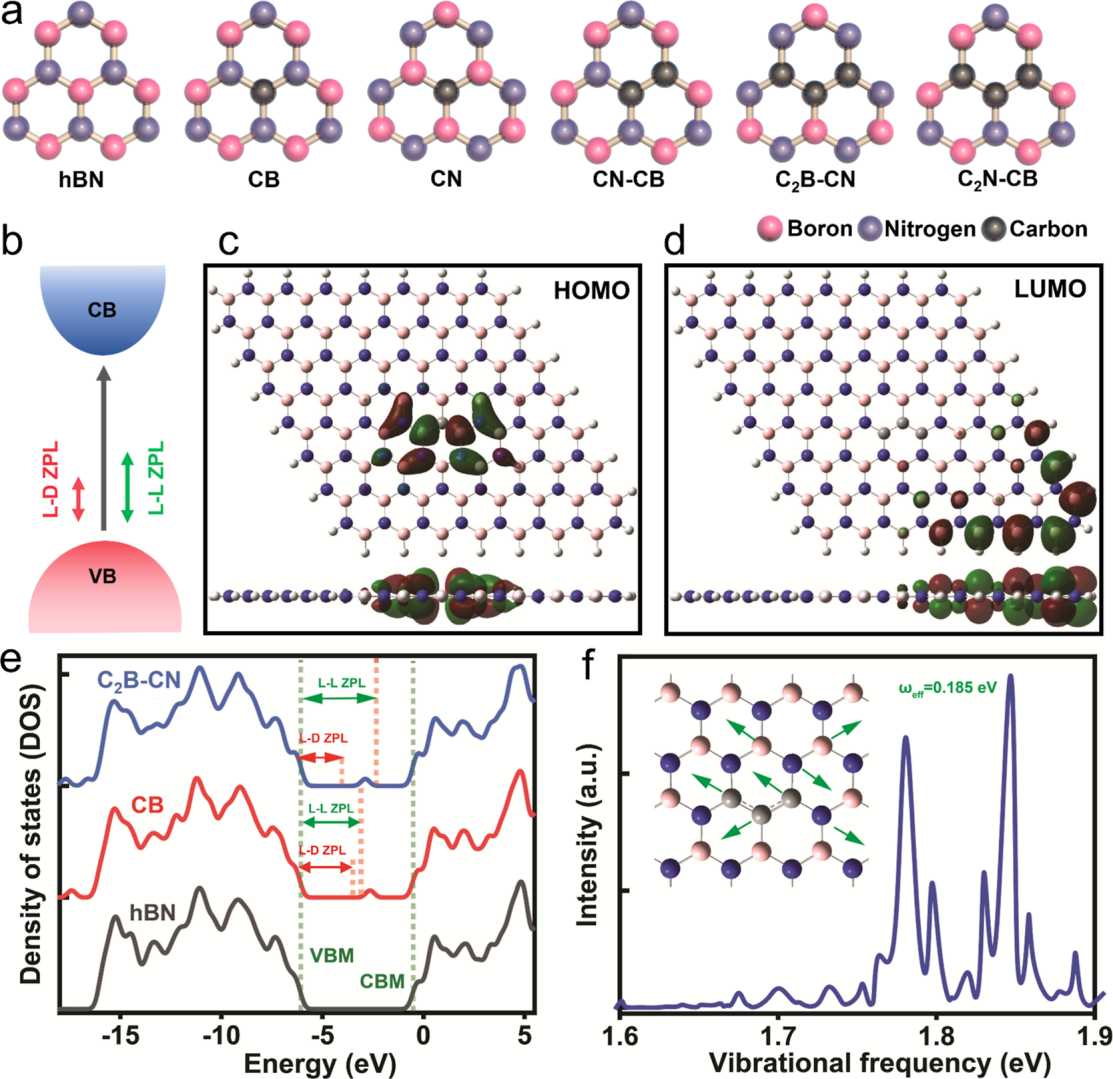
DFT calculation results of carbon-doped hBN SPEs engineering. (a) Schematic illustrations of CB, CN, CN-CB, C_2_B–CN, C_2_N-CB, CB-VN, CN-VB, C_3_B-VN, and C_3_N-VB structures. (b) Schematic of the VBM and CBM along with the frontier molecular orbitals of HOMO and LUMO involved in L–D and L–L transitions of CN–C_2_B. The frontier molecular orbitals of molecular orbital involved in L–L transitions of C_2_B–CN for the charge contribution of the (c) HOMO level and (d) LUMO level. Red and green colors represent electron availability and deficiency, respectively, with the isosurface value of 0.02 e/Å^3^. (e) Density of states (DOS) for CB, the 0.185 eV mode coupling in the zero-charged transition on C_2_B–CN. (f) Vibrational energy for the C_2_B–CN sample with the effective phonon frequency of ω_eff_ ≃ 0.185 eV. The inset is the transition displacements (green arrows) for the 0.185 eV mode coupling in the zero-charged transition on C_2_B–CN.

In order to attain a comprehensive understanding of the role of carbon defect structures in hBN for SPEs, we investigate the correlation between the atomic structure of these defects and the excitation processes that give rise to the emission phenomena. Specifically, we examine how modifications in the electronic structure of hBN, achieved by introducing carbon heteroatoms and substituting boron or nitrogen sites, impact the wavelength changes. It is important to note that the change in defect structure within the carbon-doped hBN system is expected to influence the PL wavelength through alterations in the bandgap energy and localized electronic states, thereby modulating the emission and spectral characteristics of the SPEs. To explore the underlying mechanism of the carbon-doped hBN SPE system, hybrid density functional theory (DFT) and time-dependent DFT (TD–DFT) calculations are performed. Figure S11 shows several understudied hBN structures with possible carbon defects, encompassing carbon defects (CB, CN, CN-CB, C_2_B–CN, and C_2_N-CB) and the carbon–vacancy complexes (CB-VN, CN-VB, C_2_B-VN, C_3_B-VN, C_2_N-VB, and C_3_N-VB). For each carbon defect structure, the ZPL, lattice distortion between the two configurations involved in the transition, and Huang–Rhys (HR) factor have been investigated. The HR factor, a dimensionless parameter, plays a crucial role by quantifying the coupling strength between electronic transitions and the vibrational degrees of freedom, providing insights into the vibrational dynamics and stability of SPE sites. A low HR factor in carbon point defect structures in hBN for SPE promotes higher radiative efficiency, reduces phonon-assisted relaxation, and extends the exciton lifetime, resulting in brighter, more efficient, and more stable SPE. The HR factor is calculated based on the effective phonon frequency (ω_eff_) and the lattice distortion (Δ*Q*), connecting the initial and final configurations of the emission process as follows^22^
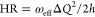
1where ω_eff_ ≃ 0.185 eV is obtained from vibrational calculations (Figure 4f). The ω_eff_ can be determined from the vibrational energy spectrum, where ω_eff_ corresponds to the value on the *x*-axis at the position of the maximum peak. The lattice distortion (Δ*Q*) is calculated by the magnitude of the mass-weighted difference in ionic displacements as follows^22^
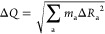
2where the sum runs over all atoms in the structure, *m*_a_ is the atomic mass of boron (= 10.811 amu), carbon (= 12.011 amu), or nitrogen (= 14.007 amu), and Δ*R*_a_ is the difference in the ionic configuration (Å) of atom a corresponding to excited and ground states. Pronounced PSBs happen normally 0.165 eV below the ZPL because of emission from the electronic excited state to the vibrationally excited electronic ground state.^22^ In order to have possible narrow band emitters, the lattice distortion (Δ*Q*) must be relatively small^22^ (for example, below 0.41 ) to result in a small HR factor (usually below 3.5).

ZPL is composed of either a localized–localized (L–L) transition, which arises from charge transfer from or into the system, or a localized–delocalized (L–D) transition, which involves the excitation of electrons from the VBM to CBM (Figure 4b). In a L–L transition, the charge transfer occurs between two localized states, typically within the same region or to the neighboring atom. On the other hand, during an L–D transition, electron excitation occurs as the electron shifts from a localized state to a delocalized one, extending its presence over a broader area encompassing multiple atoms. To obtain the L–L ZPL, the ground state geometries and energy levels of the +1, 0, and −1 charged structures are optimized using the B3LYP flavor of the DFT method with the 6-31G basis set. Table S2 shows the transition energies and structural distortions associated with different transitions involved in the L–L transitions, showing the direction of a transition. Figure S12 shows the frontier molecular orbitals of highest occupied molecular orbital (HOMO) involved in L–L transitions of charged and neutral CB, CN, C_2_B–CN, and C_2_N-CB, with the isosurface value of 0.02 e/Å^3^, indicating the presence of charge density on the carbon atoms.

In addition, to obtain the L–D ZPL, we utilized TD–DFT calculations to determine the energy levels of singlet or doublet ground and excited states for possible carbon defects. Tables 1 and S3 show the transition energies and structural distortions corresponding to different transitions involved in L–D transitions and primary electronic transitions for carbon defects and carbon–vacancy complexes, respectively. It is worth mentioning that among all the samples, only CB, CN, CN-CB, C_2_B–CN, and CB-C_2_N defect structures give rise to desired low HR factors of 3.50, 0.99, 1.49, 0.46, and 1.50, respectively, which demonstrates that they can be the active emission sites for high-quality single-photon emission. Thus, a lower HR factor correlates with greater stability in SPE, which suggests that these entities can be used as high-quality emission candidates. The structures of carbon defects that have been considered as potential candidate sites for SPE are shown in Figure 4a. For example, the L–D ZPL of 2.136 eV is predicted for CB structure with primary electronic components of HOMO → lowest unoccupied molecular orbital (LUMO), a Δ*Q* of 0.402  Å, and an HR factor of 3.50, comparable with its L–L ZPL. Besides, the L–D ZPL of 2.099 eV is predicted for the C_2_B–CN structure with primary electronic components of HOMO → LUMO, a lattice distortion (Δ*Q*) of 0.144  Å, and an HR factor of 0.46. The other samples possess a high value for the lattice distortion, which leads to high values for the HR factor. As a result, one of the possible structures for having a good SPE might be C_2_B–CN, according to the L–D ZPL of 2.099 eV (594 nm) with primary electronic components of HOMO → LUMO, a lattice distortion (Δ*Q*) of 0.144  Å, and an HR factor of 0.46. The low values for Δ*Q* and HR factors indicate a narrow photon emitter, suggesting that the emission occurs predominantly from the electronic transition within the C_2_B–CN carbon defect.

**Table 1:** Transition Energies and Structural Distortions Associated with Different Transitions Computed with B3LYP for L–D Transitions

component	ground state	ZPL (eV)	electronic transition	Δ*Q* ( Å)	HR factor
CB	doublet	2.136	HOMO→LUMO	0.402	3.50
CN	singlet	2.024	HOMO–1→HOMO	0.212	0.99
CN-CB	singlet	4.593	HOMO→LUMO	0.260	1.49
C_2_B–CN	doublet	2.099	HOMO→LUMO	0.144	0.46
C_2_N-CB	doublet	1.728	HOMO→LUMO	0.262	1.50

Figures 4c,d and S13 show the frontier molecular orbitals of the HOMO and LUMO involved in the L–D transitions of CN, CN-CB, C_2_B–CN, and CB-C_2_N, with the isosurface value of 0.02 e/Å^3^, showing their primary electronic transitions. By doing HOMO calculations, as shown in Figure 4c, we have found that after the introduction of C atoms, the charge density primarily accumulates on the defect part of the sample. This charge distribution and electronic configuration are due to the higher electronegativity of carbon (2.55) compared to boron (2.04), which accumulates the charge around the defect. This accumulation of charge leads to the localization of wavefunction, changing the emission wavelength of the SPEs toward a pure SPE. As shown in Figure 4e, the DOS suggests the presence of L–D and L–L ZPL points between VBM and CBM (CBM–VBM = 5.77 eV). This also suggests that introducing carbon atoms can enable alignment of the L–D, which is of fundamental importance to achieve a favorable L–D ZPL. In addition, the DOS for CB and C_2_B–CN possesses a distinct peak around −2 eV, which is due to the presence of π* antibonding orbitals after the introduction of C atoms to the hBN. This indicates that π* antibonding orbitals play an important role in the L–D ZPL.^48^Figures 4f and S14 show the vibrational frequency for the C_2_B–CN sample with the effective phonon frequency of ω_eff_ ≃ 0.185 eV. The inset is the transition displacements (green arrows) for the 0.185 eV mode coupling in the zero-charged transition on C_2_B–CN. The calculation for the effective phonon frequency ω_eff_ of CB is depicted in Figure S14. Analysis of the XPS results reveals that the carbon–boron (CB) portion is greater than the carbon–nitrogen (CN) portion (CB > CN). As a result, one possible conversion of defect structures is proposed, from CB to C_2_B–CN, which exhibits a L–D ZPL shift from 2.136 and 2.099 eV, where the primary electronic transitions involved in this structure are HOMO → LUMO. Additionally, the lattice distortions (Δ*Q*) are measured to be 0.402 Å and 0.144  Å, respectively, and the HR factor is found to be 3.5 and 0.46 for CB and C_2_B–CN, respectively. The small values of Δ*Q* and HR indicate that the emitter possesses a narrow emission linewidth, making it a promising candidate for a highly efficient photon emitter. CB and C_2_B–CN exhibit favorable HR attributes for both L–L and L–D transitions, implying the feasibility of both mechanisms within these defect configurations. Notably, a consistent observation emerges where the ZPL is lower during L–D transitions compared to L–L transitions for both CB and C_2_B–CN, suggesting a higher probability for the L–D transition to function as the predominant pathway for SPE. In particular, both CB^24,29,49^ and C_2_B–CN^29,49−51^ defects in hBN have been reported as the potential single-photon emission sites. These findings offer compelling evidence and demonstrate a strong agreement between the results obtained from DFT calculations and experimental observations.

## Conclusions

In conclusion, a strategy of carbon defect engineering in hBN for SPEs’ wavelength modulation was developed. This entails employing molten copper substrates during CVD synthesis to ensure a homogeneous continuous carbon supply before the formation of a perfect hBN flake, thereby enabling a high carbon doping level while maintaining a uniform distribution of emission sites. By systematically varying the carbon concentration in Cu substrates from 0.0005 at % to 0.082 at %, we achieve precise defect structure engineering in hBN, transitioning from CB to C_2_B–CN defect structures and enabling precise control over carbon incorporation. The modification of the point defect structure in carbon-doped hBN results in changes in its electronic structure, leading to alterations in the wavelength of both the excitation and emission processes. Hybrid DFT and TD–DFT calculations are utilized to examine the ZPLs, L–L and L–D transitions, lattice distortions between the involved configurations, and HR factors for carbon defects and carbon–vacancy complexes. Remarkably, the observed SPE data has exhibited strong agreement with the results obtained from DFT calculations, XPS, and Raman spectroscopy, providing comprehensive validation of the achieved defect engineering and its impact on the optical properties of hBN. This work constitutes a substantial progress in defect engineering for quantum emission in 2D materials, marking a significant advancement toward the development of on-chip devices.

## Experimental Section/Methods

### Material Growth

2 mg portion of ammonia borane (AB) powder (Aladdin, 97%) was placed in a quartz boat and used as the precursor for boron and nitrogen sources. A piece of 15 mm× 15 mm Cu foil with different carbon-containing concentrations (0.0005 atom %, 0.003 atom %, and 0.082 atom %) was washed in acetic acid for 5 min for removing the impurity and oxide layer, rinsed with deionized water, and finally dried by argon gun. The Cu foil was stacked on tungsten foil and loaded into 1 in. tube furnace. The AB powder was placed 32 cm upstream from the Cu/W foil and heated by a heating coil for thermal dehydration during CVD growth. The tube was purged with 250 sccm of argon gas for 10 min before growth. Then, the furnace was ramped up to 1090 °C in 40 min under a gas mixture of 250 sccm Ar gas and 25 sccm H_2_ gas and then kept 10 min for ensuring the complete melting of the Cu foil. To start the hBN growth, the AB powder was heated to 90 °C for certain duration, 30 min for isolated hBN grain growth and 2.5 h for continuous film growth. The heating coil was turned off, and the hydrogen gas flow was stopped. The system was quickly cooled to room temperature under an argon atmosphere. After growth, the Cu/W foil was heated up to 180 °C on a hot plate (2 min) to oxide the Cu surface for visualizing the hBN grains.

### Transfer of the hBN Samples

The hBN samples were transferred by using a technique called PMMA-assisted bubble transfer. First, the required hBN on a copper foil was appropriately sized by cutting it. The hBN samples were then spin-coated with PMMA at 500 rpm for 10 s, followed by 5000 rpm for 50 s. The samples were utilized as the cathode, while a platinum (Pt) foil functioned as the anode, which were connected to a direct current power supply. An electrolyte solution of 1 M sodium hydroxide (NaOH) was prepared. By application of a voltage of 5 V, hydrogen bubbles were generated, gradually separating the hBN/PMMA films from the Cu substrates. Then, the hBN/PMMA films were transferred and allowed to float on deionized water to eliminate any adsorbed ions. Afterward, the hBN/PMMA films were collected using a SiO_2_/Si wafer and dried on a hot plate at a temperature of 75 °C for a duration of 5 min. Finally, the PMMA layer was removed by immersing the sample in 60 °C acetone for 1 h.

### Characterizations

The hBN samples were immediately seen under an optical microscope (LEICA DMLM optical microscope). Raman spectroscopy using a 514 nm source and a Renishaw Raman RM3000 scope revealed a macroscopical structure. AFM was used to measure the thickness of hBN crystals (AFM, Bruker Innova, laser diode 670 nm, Class b). For element-state analysis, X-ray photoelectron spectroscopy (XPS, Thermal Fisher, ESCALAB 250Xi) was carried out. To perform TEM characterization, the samples were transferred onto TEM grids using the bubble transfer method, assisted by PMMA. After removal of PMMA, the sample was then annealed under 350 °C and 200 sccm argon gas for 3 h. TEM was measured using a JEM 2010F microscope operated at a voltage of 200 kV.

### Photoluminescence Measurements

Using a specialized scanning confocal microscope and a continuous-wave (CW) 532 nm laser, the spectra of confocal PL were carried out (Gem 532, Laser Quantum Ltd.). A high numerical aperture (100, NA = 0.9, Nikon) objective lens was used to focus the laser, and an X–Y piezo fast steering mirror (FSM-300) was used for the scanning image. Before coupling into a graded-index multimode fiber (0.22 NA) with a fiber aperture of 62.5 μm functioning as a confocal pinhole, the collecting pathway was conducted through filtration utilizing a 532 nm dichroic mirror (532 nm laser BrightLine, Semrock). The emission was directed toward two avalanche photodiodes (Excelitas Technologies) arranged in a Hanbury-Brown and Twiss configuration or toward a spectrometer (Princeton Instrument Inc., Acton Spectra Pro) using a flip mirror.

### DFT and TD–DFT Calculations

Our hybrid DFT calculations were preformed based on the Becke 3-parameter, Lee, Yang, and Parr (B3LYP) functional 6-31G^52,53^ basis set for ground state optimizations of charged systems and calculation of L–D ZPL.^54−56^ Moreover, we calculated the energy levels of the singlet or doublet excitons using TD–DFT to evaluate the L–L ZPL based on the optimized excited state geometries. Previous studies have verified that hybrid B3LYP functional can precisely depict bandgap energy along with exciton energies and charge transfer.^57−61^ For example, the bandgap energy of hBN is predicted to be 5.77 eV using B3LYP functional, which is in agreement with the theoretical bandgap of 5.71 eV and experimental bandgap of 6.11 eV reported in the literature.^22,23^ We also applied the DFT-D3 method to include van der Waals attraction (London dispersion) interactions.^62,63^ The Gaussian 16 software was utilized for performing all the DFT calculations, and visualizations are performed using GaussView 6.0.16 and Multiwfn 3.8.^64,65^

## Supporting Information Available

Details about the experimental process from hBN growth to SPE measurement; optical images and AFM analysis of the hBN grain grown from Cu foil with different carbon concentrations; Raman and XPS spectra of high-purity exfoliated hBN; SPE spectra obtained from different regions of the samples grown on various copper foils; distributions of *E*_2g_ Raman peak positions of hBN growth from 0.003% C–Cu; Raman intensity mapping of the hBN samples grown from different carbon-containing coppers; TEM–EDS mapping of hBN growth from 0.003% carbon-containing Cu; optical images of the hBN films transferred onto the SiO_2_/Si wafer, second-order correlation histogram g^(2)^(τ) analysis; optimized ground state structure of possible carbon defect in hBN; frontier molecular orbitals of molecular orbital involved in L–L and L–D transitions; vibrational energies for the CB and C_2_B–CN defects; ICP–MS analysis of Cu foils with different carbon concentrations; and computational analysis of transition energies and structural distortions associated with L–L and L–D transitions (PDF)
