# Supplementary material for: Structured-Defect Engineering of Hexagonal Boron Nitride for Identified Visible Single-Photon Emitters
Source: ACS Nano. 2025 Feb 28;19(9):8509–19. doi: 10.1021/acsnano.4c11413 (PMC11912567; doi:10.1021/acsnano.4c11413)
Supplement: Supplementary file 1 — nn4c11413_si_001.pdf [file nn4c11413_si_001.pdf]

Supporting Information

# Structured-Defect Engineering of Hexagonal Boron Nitride for Identified Visible Single Photon Emitters

Tsz Wing Tang[a#], Ritika Ritika[b,c#], Mohsen Tamtaji[a,d#], Hongwei Liu[a], Yunxia Hu[a], Zhenjing Liu[a], Patrick Ryan Galligan[a], Mengyang Xu[a], Jinghan Shen[a], Jun Wang[a], Jiawen You[a], Yuyin Li[a], GuanHua Chen[d,e*], Igor Aharonovich[b,c*], Zhengtang Luo[a*]

[a]*Department of Chemical and Biological Engineering, The Hong Kong University of Science and Technology, Hong Kong SAR 999077, P. R. China;*

[b]*School of Mathematical and Physical Science, University of Technology Sydney, Ultimo, New South Wales 2007, Australia;*

[c]*ARC Centre of Excellence for Transformative Meta-Optical Systems, Faculty of Science, University of Technology Sydney, Ultimo, New South Wales 2007, Australia;*

[d]*The Hong Kong Quantum AI Lab Limited, Hong Kong SAR 999077, China*

[e]*Department of Chemistry, The University of Hong Kong, Hong Kong SAR 999077, China*

[#]*These authors contributed equally to this work.*

*E-mail: keztluo@ust.hk.

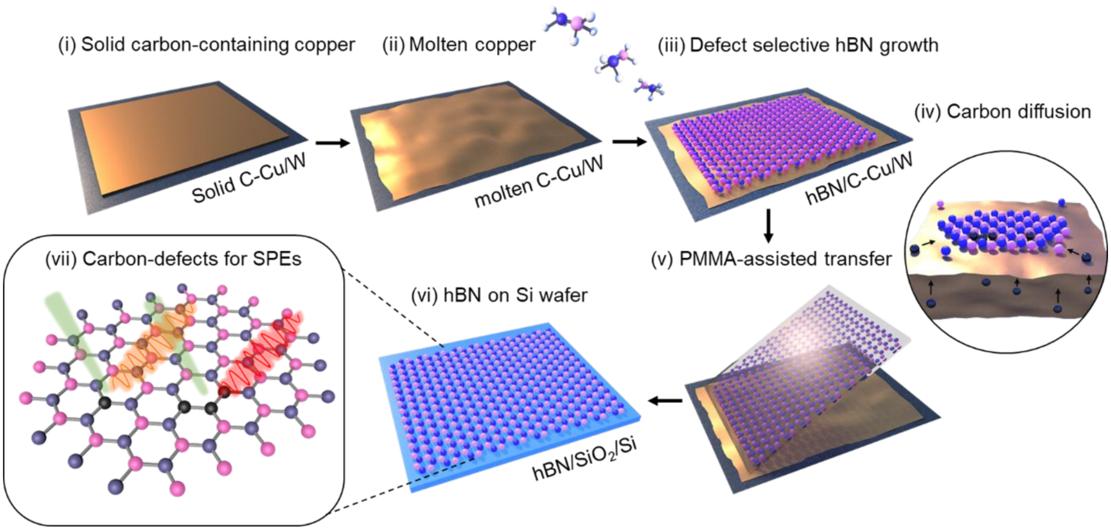


**Figure S1.** Illustration of the experimental process from hBN growth to SPEs measurement.

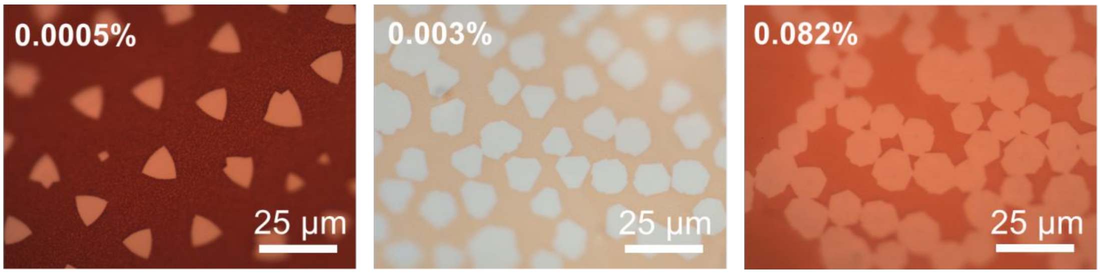


**Figure S2.** Optical images of the hBN grains grown from Cu foils with different carbon concentrations: 0.0005%, 0.003%, and 0.082%.

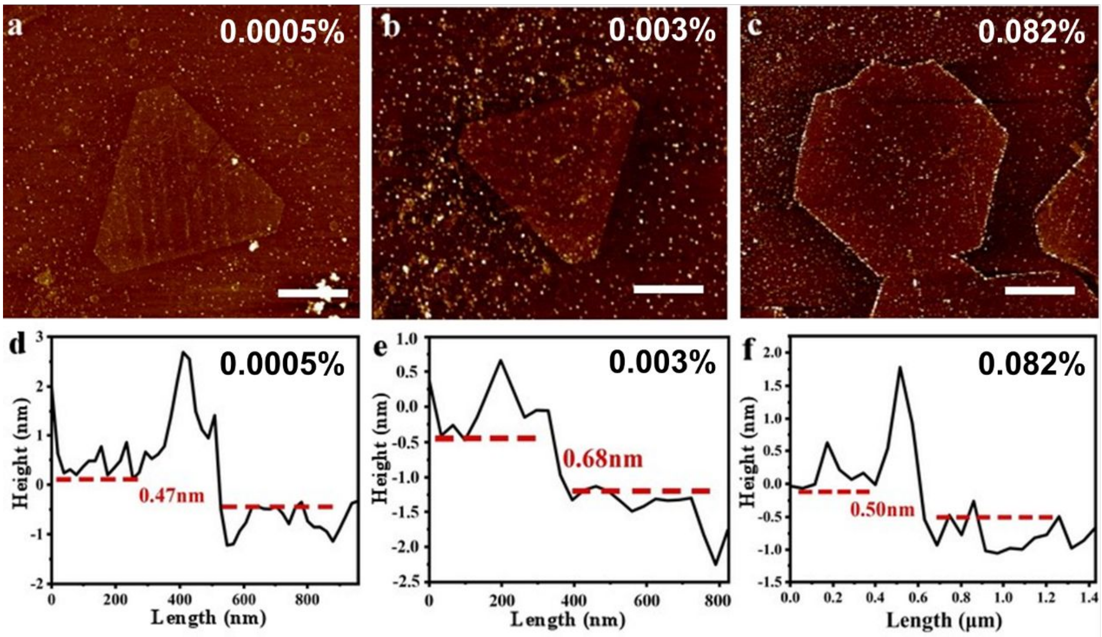


**Figure S3.** AFM measurements and the corresponding line profiles were conducted on hBN samples grown from different carbon containing coppers; (a, d) 0.0005%, (b, e) 0.003%, and (c, f) 0.082%. In all cases, the hBN crystals were found to be monolayers (thickness about 0.33 nm), indicates the high quality of the as-grown monolayer hBN.

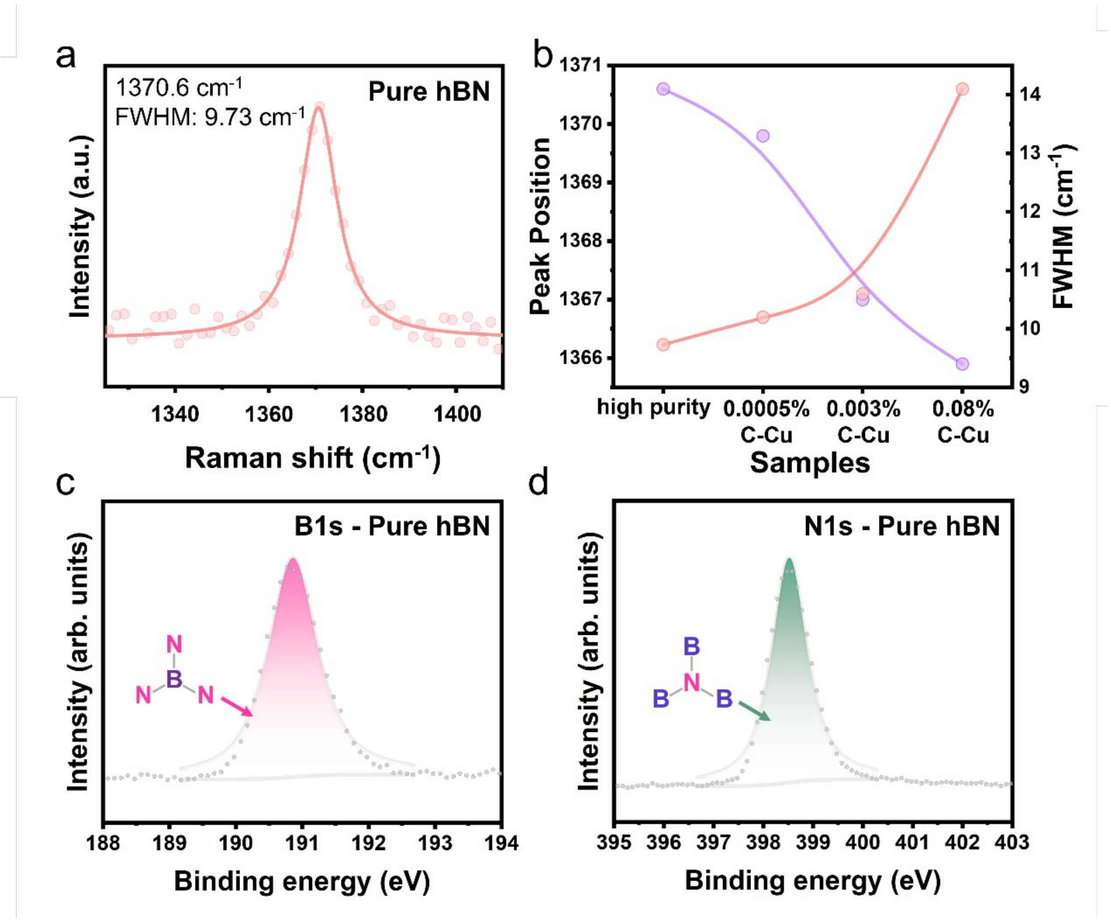


**Figure S4.** (a) The Raman spectrum of pure monolayer sample exfoliated from bulk hBN sample, and (b) the peak positions and FWHM values of the $E_{2g}$ mode from the hBN samples growth from various growth cases and high purity hBN exfoliated from bulk. the XPS spectra of pure hBN obtained from exfoliated bulk at the (c) B1s and (d) N1s core levels.

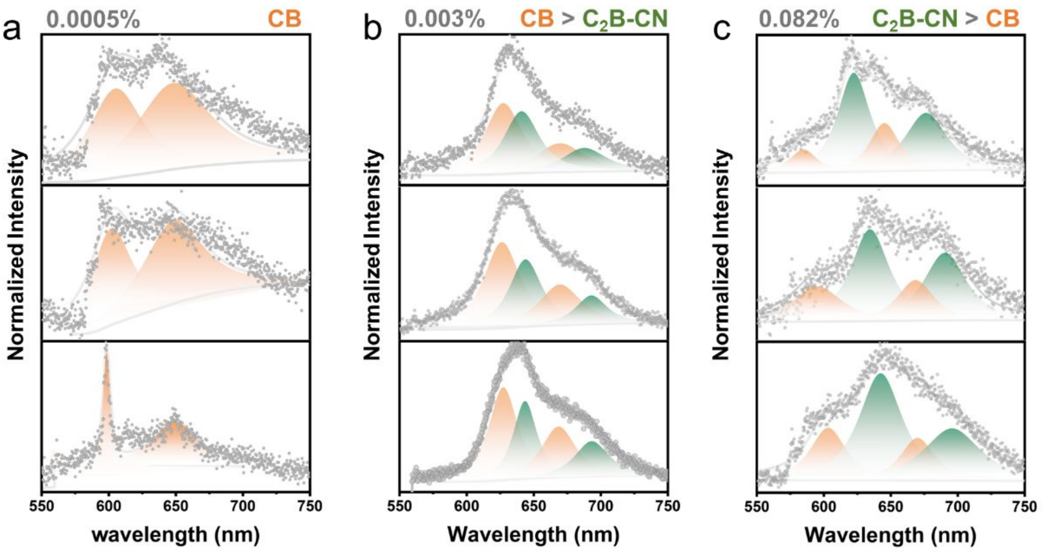


**Figure S5.** The SPE spectra obtained from different regions of the same samples as depicted in **Figure 1g-i**, grown on various copper foils with (a) 0.0005%, (b) 0.003%, and (c) 0.082% carbon concentrations.

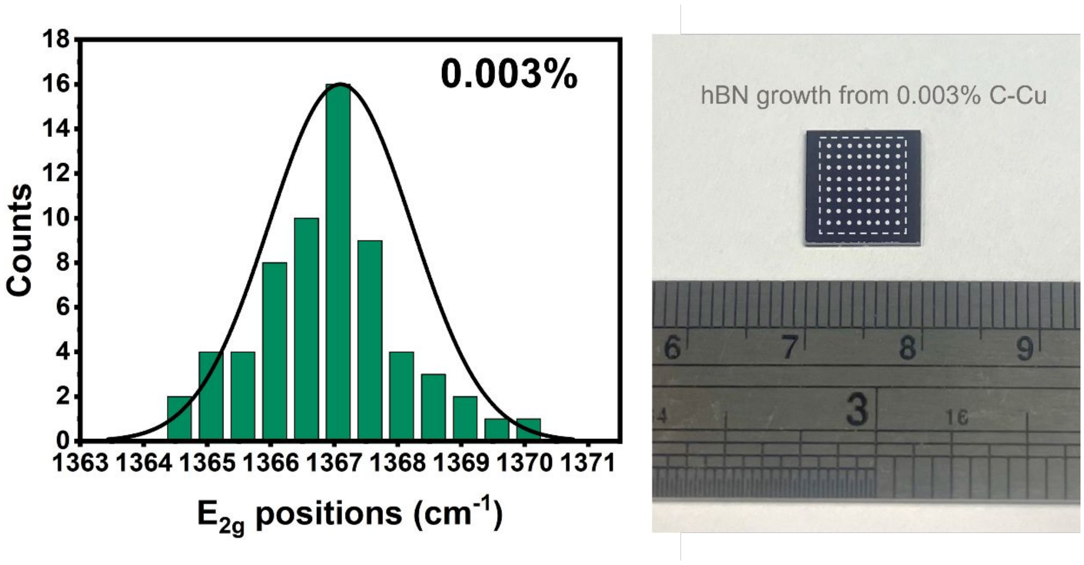


**Figure S6.** Distributions of $E_{2g}$ Raman peak positions measured from 8 × 8 data sets (64 points) of hBN growth from 0.003% C-Cu.

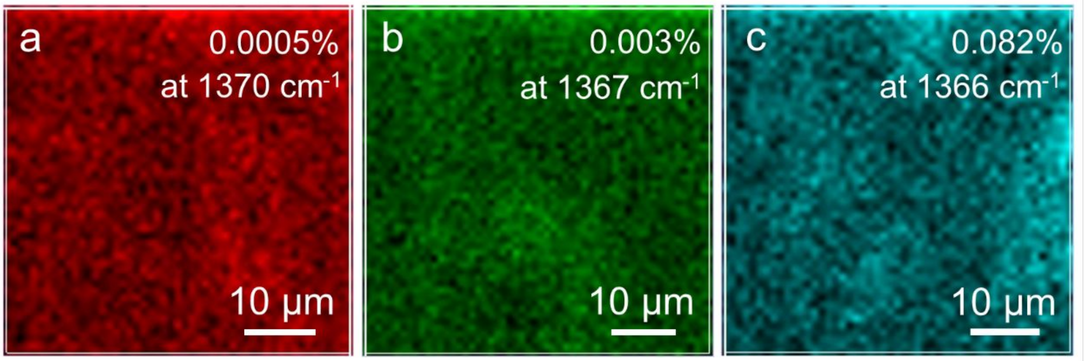


**Figure S7.** Intensity mapping of the Raman $E_{2g}$ band for carbon-doped hBN samples grown from different carbon containing coppers; (a) 0.0005%, (b) 0.003%, and (c) 0.082%.

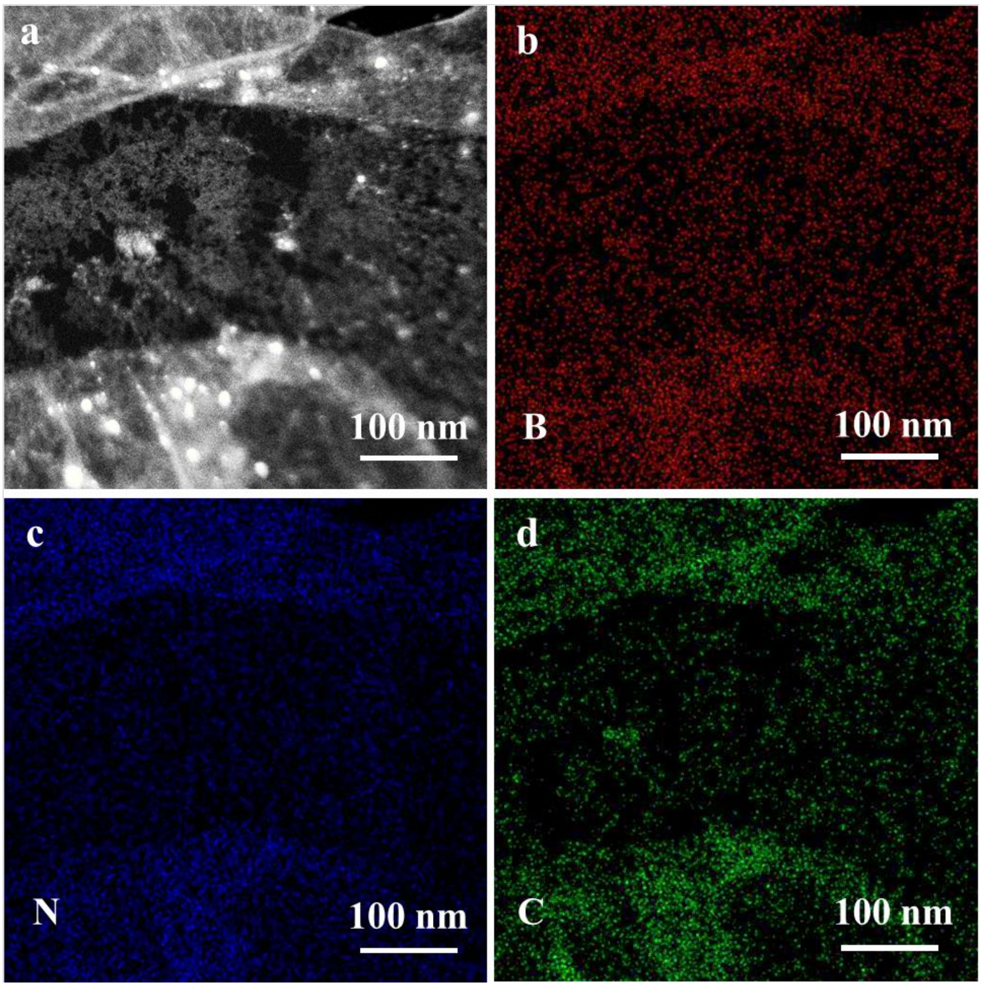


**Figure S8.** The corresponding EDS mapping of hBN film growth from 0.003% carbon-containing Cu foil shown in **Fig. 3d-e**, with uniform elemental distribution of both B, N, and C.

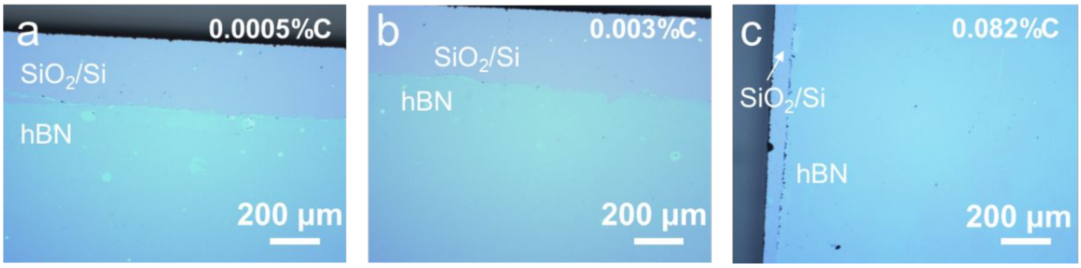


**Figure S9.** Optical images of the as-grown hBN films from (a)0.0005%, (b) 0.003% and (c)0.082% carbon-containing Cu foils, and transferred on $SiO_2$/Si wafer for subsequence SPE measurements.

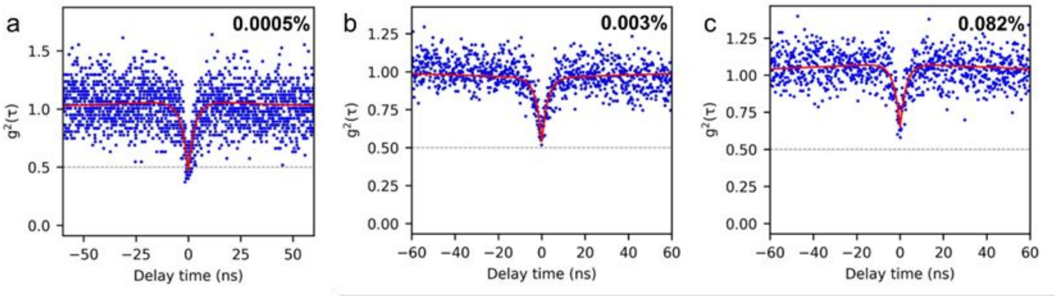


**Figure S10.** $g^{(2)}$ $(\tau)$ recorded from each SPE in **Figure 1g-i**. The second-order correlation histogram ($g^{(2)}(\tau)$) was measured for a representative emitter within the irradiated region. The measurement encompassed the entire emission spectrum and was conducted using a Hanbury Brown and Twiss configuration. The measured emissions in each case provide confirmation of the quantum nature of the emitted photons.

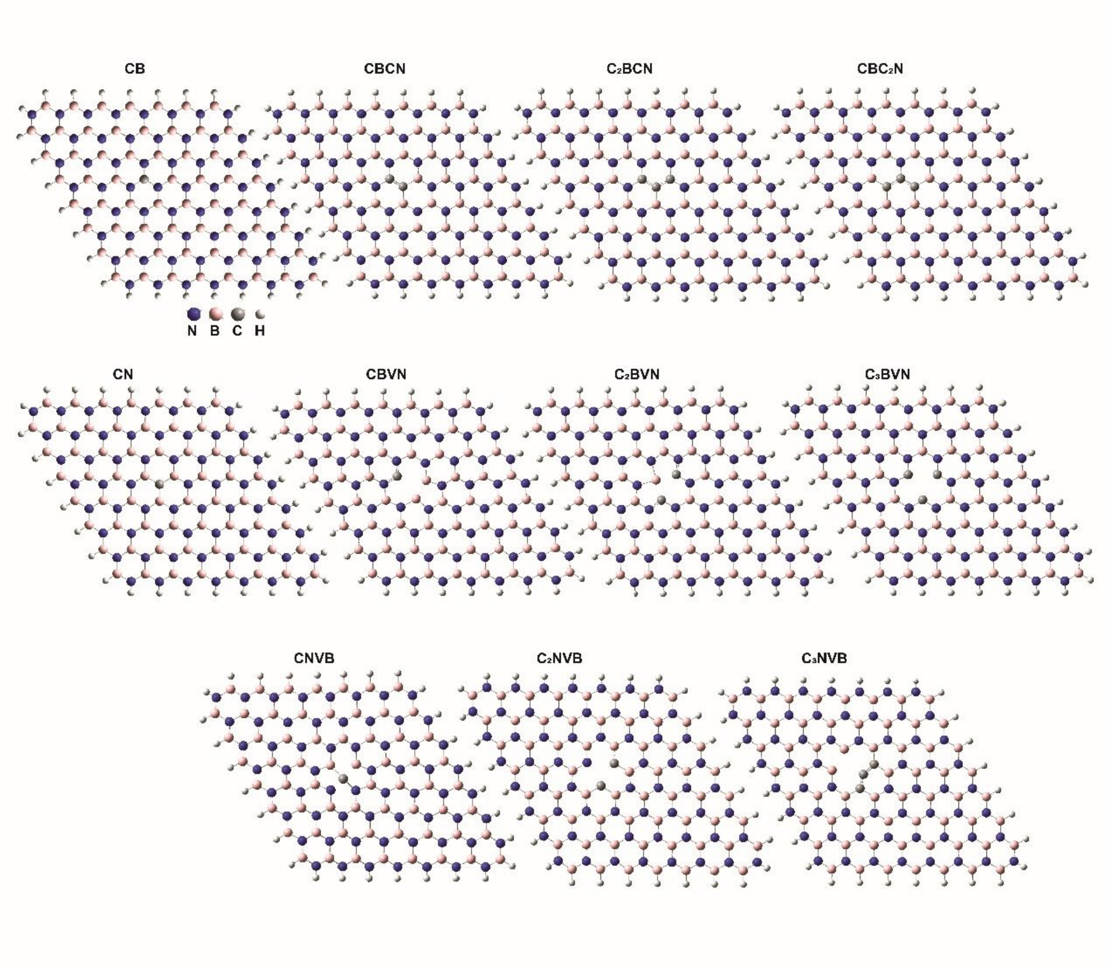


**Figure S11.** Optimized ground state structure of CB, CN, CN-CB, $C_2$B-CN, $C_2$N-CB, CB-VN, CN-VB, $C_2$B-VN, $C_2$N-VB, $C_3$B-VN, and $C_3$N-VB samples from DFT calculation at the level of B3LYP and 6-31G basis set using Gaussian 16.

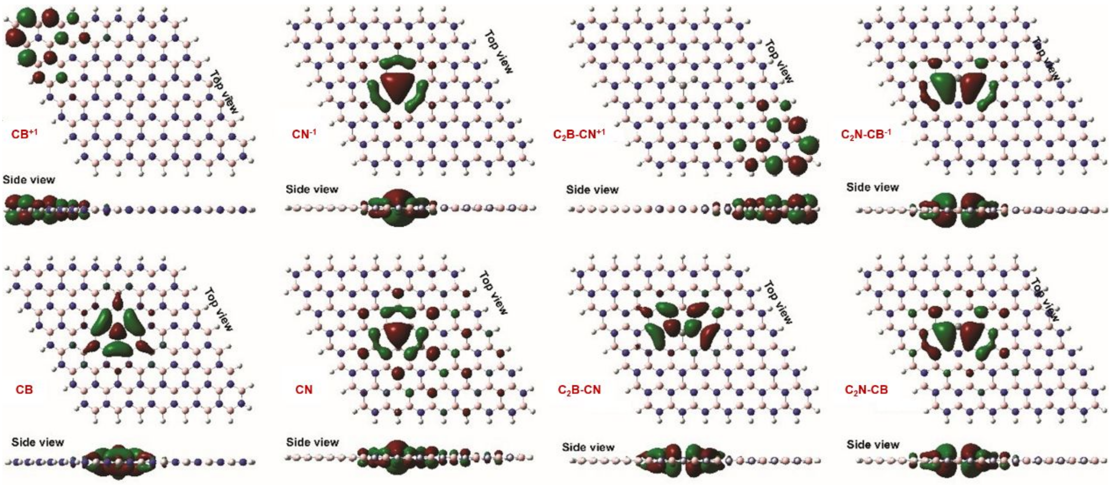


**Figure S12.** Frontier molecular orbitals of molecular orbital involved in L-L transitions of CB, CN, $C_2B$-CN, and $C_2N$-CB, with the isosurface value of 0.02 e/Å$^3$, indicates the presence of charge density on the carbon atoms. (from DFT calculation at the level of B3LYP and 6-31G basis set using Gaussian 16). Red and green colors represent electron availability and deficiency, respectively, with the isosurface value of 0.02 e/Å$^3$.

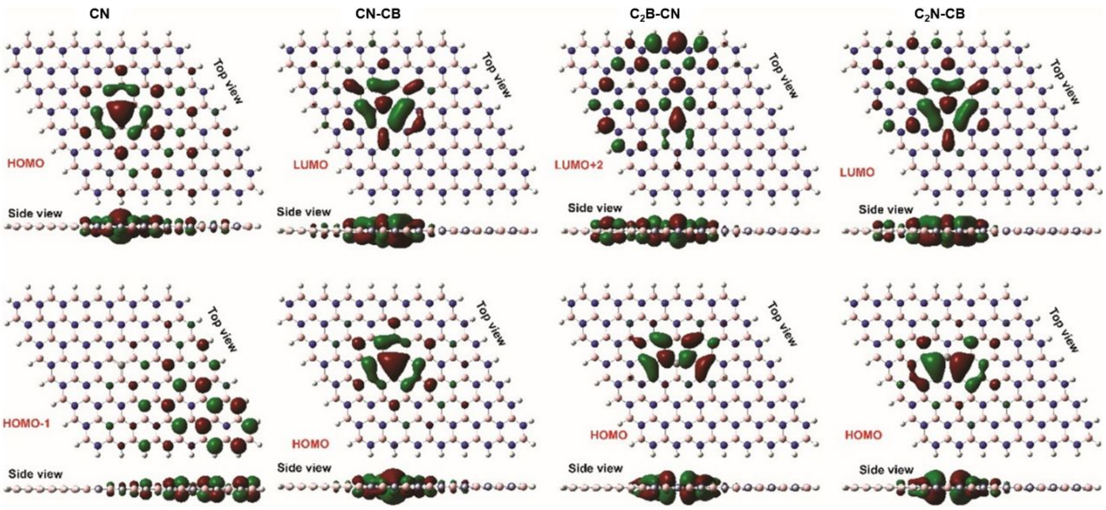


**Figure S13.** Frontier molecular orbitals of molecular orbitals involved in L-D transitions of CN, CN-CB, $C_2B$-CN, and $C_2N$-CB, with the isosurface value of 0.02 e/Å$^3$ (from DFT calculation at the level of B3LYP and 6-31G basis set using Gaussian 16). Red and green colors represent electron availability and deficiency, respectively, with the isosurface value of 0.02 e/Å$^3$.

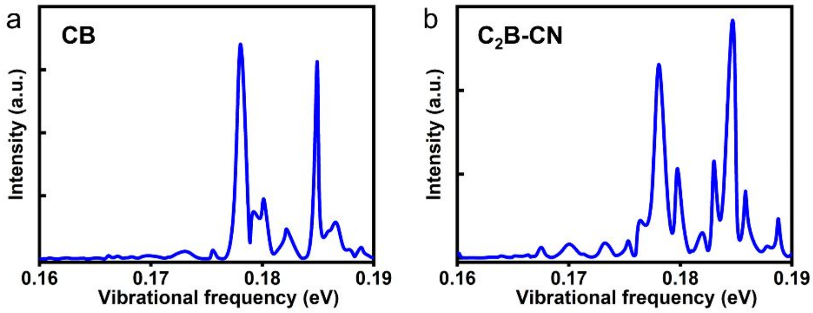


**Figure S14.** The vibrational energies for the (a) CB and (b) $C_2$B-CN defects with the effective phonon frequency of $\omega_{eff} \simeq 0.178$ eV and $\omega_{eff} \simeq 0.185$ eV, respectively.

**Table S1.** Inductively coupled plasma mass spectrometry (ICP-MS) result.

| | S | O | C | Al | P | Cr | Fe | Ni | Zn | Ag | Sn | Sb | Pb | Bi |
|---|---|---|---|---|---|---|---|---|---|---|---|---|---|---|
| **Cu #1** | 0.0010 | 0.0009 | 0.0005 | 0.0001 | 0.0014 | 0.0010 | 0.0011 | 0.0002 | 0.0001 | 0.0004 | 0.0003 | <0.0000 | 0.0004 | 0.0001 |
| **Cu #2** | 0.0010 | 0.0010 | 0.0030 | 0.0001 | 0.0030 | <0.0000 | 0.0032 | 0.0112 | 0.0055 | 0.0027 | 0.0133 | 0.0002 | 0.0010 | 0.0001 |
| **Cu #3** | 0.0009 | 0.0008 | 0.0820 | 0.0001 | 0.0036 | <0.0000 | 0.0039 | 0.0136 | 0.0066 | 0.0029 | 0.0150 | 0.0002 | 0.0012 | 0.0001 |

The Cu foils with different carbon concentrations were dissolved in nitric acid to conduct ICP-MS for determine the atomic percentages of carbon impurities in Cu foils.

**Table S2.** Transition energies and structural distortions associated with different transitions computed with B3LYP for localized-localized (L-L) transitions. The ZPL corresponding to the transition involving the VB edge ($ZPL_{VBM}$). Arrows indicate the direction of a transition. ZPL, ΔQ, and HR stand for zero-phonon line, the lattice distortion between the two defect configurations involved in the transition, and Huang-Rhys factor.

| **Component** | **Charge transfer** | **$ZPL_{VBM}$ (eV)** | **ΔQ ($\sqrt{amu}$ Å)** | **HR factor** |
|---|---|---|---|---|
| CB | 0→+1 | 3.40 | **0.237** | 1.24 |
| CN | -1→0 | 2.86 | **0.220** | 1.06 |
| CN-CB | 0→+1 | 6.57* | 0.186 | -- |
| $C_3$B-VN | 0→+1 | 2.85 | 0.968** | -- |
| $C_3$B-VN | -1→0 | 2.68 | 16.113** | -- |
| $C_2$B-VN | 0→+1 | 4.15 | 0.466** | -- |
| $C_2$B-VN | -1→0 | 2.34 | 0.489** | -- |
| $C_2$B-CN | 0→+1 | 3.65 | **0.266** | 1.56 |
| CB-$C_2$N | -1→0 | 2.84 | **0.224** | 1.10 |
| $C_2$N-CB | 0→+1 | 6.54* | 0.159 | -- |
| CB-VN | 0→+1 | 4.74 | 0.884** | -- |
| $C_2$N-VB | 0→+1 | 1.00 | 2.461** | -- |
| $C_2$N-VB | -1→0 | 3.23 | 0.680** | -- |
| CN-VB | 0→+1 | 7.26* | 24.404** | -- |
| CN-VB | -1→0 | 4.06 | 8.869** | -- |
| $C_3$N-VB | 0→+1 | 5.80* | 1.115** | -- |
| $C_3$N-VB | -1→0 | 0.49 | 0.360 | -- |

*Out of bandgap range (5.77 eV)

**ΔQ is too high. The ΔQ lower than 0.41 $\sqrt{amu}$ Å is considered to possess a possible narrow band emitter.

Among all the atomic structures, only CB, CN, $C_2$B-CN, and $C_2$N-CB satisfy desired HR factors of 1.24, 1.06, 1.56, and 1.10 for excellent single photon emitters. The other samples, especially the ones with vacancies with high vibrational modes, either possess the ZPL of out of bandgap range (5.77 eV) or high value for the lattice distortion ($\Delta Q$ > 0.41 $\sqrt{amu}$ Å), leading to high HR factor values which are not suitable for the SPEs. The ZPL of 3.40 eV has been predicted for CB structure with the charge transfer from 0 to +1, the lattice distortion ($\Delta Q$) of 0.237 $\sqrt{amu}$ Å, and Huang-Rhys factor (HR) of 1.24, in agreement with the literature.[1, 2] And the ZPL of 2.86 eV is predicted for CN structure with the charge transfer from -1 to 0, the lattice distortion (ΔQ) of 0.220 $\sqrt{amu}$ Å, and Huang-Rhys factor (HR) of 1.06.

**Table S3.** Transition energies and structural distortions of the carbon-vacancy complex defects associated with different transitions computed with B3LYP for localized-delocalized (L-D) transitions.

| Component | Ground state | ZPL (eV) | Electronic transition | ΔQ ($\sqrt{amu}$ Å) | HR factor |
|---|---|---|---|---|---|
| **$C_3$B-VN** | singlet | 1.315 | HOMO→LUMO+3 | 0.848** | -- |
| **$C_2$B-VN** | doublet | 1.731 | HOMO→LUMO | 1.188** | -- |
| **CB-VN** | singlet | 1.707 | HOMO→LUMO | 0.904** | -- |
| **$C_2$N-VB** | doublet | 0.714 | HOMO→LUMO+1 | 0.895** | -- |
| **CN-VB** | singlet | 4.057 | HOMO→LUMO | 0.905** | -- |
| **$C_3$N-VB** | singlet | 2.402 | HOMO→LUMO | 0.588** | -- |

**$\Delta Q$ is too high. The $\Delta Q$ lower than 0.41 $\sqrt{amu}$ Å is considered to possess a possible narrow band emitter.

**References**

1. Linderälv, C.; Wieczorek, W.; Erhart, P. Vibrational signatures for the identification of single-photon emitters in hexagonal boron nitride. *Physical Review B* **2021,** *103* (11).

2. Weston, L.; Wickramaratne, D.; Mackoit, M.; Alkauskas, A.; Van de Walle, C. G. Native point defects and impurities in hexagonal boron nitride. *Physical Review B* **2018,** *97* (21).
